# The Relative Role of Toxins A and B in the Virulence of *Clotridioides difficile*

**DOI:** 10.3390/jcm10010096

**Published:** 2020-12-30

**Authors:** Andrew M. Skinner, S. Tyler Phillips, Michelle M. Merrigan, Kevin J. O’Leary, Susan P. Sambol, Farida Siddiqui, Lance R. Peterson, Dale N. Gerding, Stuart Johnson

**Affiliations:** 1 Research Service, Edward Hines Jr., Veterans Affairs Hospital, Infectious Disease Section, Hines, IL 60141, USA; andrew.skinner@lumc.edu (A.M.S.); Stylerphillips@gmail.com (S.T.P.); michelle.m.merrigan@gmail.com (M.M.M.); susan.sambol@va.gov (S.P.S.); siddiqui_farida@yahoo.com (F.S.); dale.gerding2@va.gov (D.N.G.); 2 Department of Medicine, Loyola University Medical Center, Chicago, IL 60153, USA; 3 Department of Medicine, Northwestern University Medical School, Chicago, IL 60611, USA; keoleary@nmh.org; 4 Pritzker School of Medicine, University of Chicago, Chicago, IL 60637, USA; lancerp@comcast.net

**Keywords:** *Clostridioides difficile*, molecular epidemiology, toxinotyping, restriction endonuclease analysis, toxin A, toxin B

## Abstract

Most pathogenic strains of *C. difficile* possess two large molecular weight single unit toxins with four similar functional domains. The toxins disrupt the actin cytoskeleton of intestinal epithelial cells leading to loss of tight junctions, which ultimately manifests as diarrhea in the host. While initial studies of purified toxins in animal models pointed to toxin A (TcdA) as the main virulence factor, animal studies using isogenic mutants demonstrated that toxin B (TcdB) alone was sufficient to cause disease. In addition, the natural occurrence of TcdA−/TcdB+ (TcdA−/B+)mutant strains was shown to be responsible for cases of *C. difficile* infection (CDI) with symptoms identical to CDI caused by fully toxigenic (A+/B+) strains. Identification of these cases was delayed during the period when clinical laboratories were using immunoassays that only detected TcdA (toxA EIA). Our hospital laboratory at the time performed culture as well as toxA EIA on patient stool samples. A total of 1.6% (23/1436) of all clinical isolates recovered over a 2.5-year period were TcdA−/B+ variants, the majority of which belonged to the restriction endonuclease analysis (REA) group CF and toxinotype VIII. Despite reports of serious disease due to TcdA−/B+ CF strains, these infections were typically mild, often not requiring specific treatment. While TcdB alone may be sufficient to cause disease, clinical evidence suggests that both toxins have a role in disease.

## 1. Introduction

Most virulent strains of *Clostridioides difficile* produce two large single unit toxins which act via glycosylation of small GTP-binding proteins involved in cell cytoskeleton organization resulting in the disruption of epithelial tight junctions [1,2,3]. These two primary toxins, identified as toxin A (TcdA) and toxin B (TcdB), are encoded by *tcdA* and *tcdB* located with the pathogenicity locus [4]. As such, the detection of toxins A and B in patient stool specimens has been utilized by clinical laboratories to diagnose *C. difficile* infection (CDI) for decades [5]. The first toxin assay available was a cell culture cytotoxin assay, which primarily reflects the cytopathic effects of TcdB on eukaryotic cell culture [6]. In the late 1990s, many clinical laboratories implemented TcdA enzyme immunoassays (EIA) for *C. difficile* testing in place of cytotoxin assays because of the rapidity and simplicity of the test, as well as initial data showing comparable results with cell culture assays [7,8]. These EIAs employed a monoclonal antibody (PCG-4) that recognizes epitopes encoded by a region of highly repetitive DNA sequences in the 3′ end of *tcdA* [9]. Previous studies of purified toxins in animal models indicated that TcdA was the primary virulence determinant; therefore, TcdA was included as the sole target antigen in these early immunoassays [10]. Subsequent studies identified the importance of TcdB, including animal studies with challenges by isogenic *tcdA* and *tcdB* mutant strains, which demonstrated that TcdB alone could result in disease [11,12].

*C. difficile* toxin variants have been described that do not react with TcdA EIA but are cytotoxic in cell culture assays [13]. These strains share common genetic alterations including truncation at the 3′ end of *tcdA* and a point mutation at the 5′ end of *tcdA* that introduces a premature stop codon at amino acid position 47 [13,14]. CDI due to these toxin A−/B+ (TcdA−/B+) variants has been increasingly common across the world, including multiple nosocomial outbreaks, highlighting the limitations of TcdA EIA as a diagnostic tool [15,16,17,18,19,20,21,22,23]. These limitations were further highlighted by a fatal case of pseudomembranous colitis due to a TcdA−/B+ variant strain (REA Type CF4) for which the diagnosis of CDI was delayed by reliance on the TcdA EIA test which was repeatedly negative [24].

*C. difficile* testing has evolved over the past 20 years due to detection limitations and the inability of TcdA EIAs to detect *C. difficile* TcdA−/B+ variant strains and TcdA EIA has been replaced by EIAs that detect both toxins. However, still unanswered is the clinical and epidemiologic significance of TcdA−/B+ variant *C. difficile* isolates. In this study, we investigated the incidence, outcomes, and laboratory characterization of TcdA−/B+ variant CDI.

## 2. Experimental Section

We conducted a single-center retrospective study from September 1999 to January 2002 at Northwestern Memorial Hospital located in Chicago, Illinois to determine the incidence, clinical impact, and the molecular epidemiology of CDI related to TcdA−/B+ variant isolates. 

### 2.1. Clinical Microbiology Laboratory Testing

From September 1999 to January 2002, patient stool samples were submitted to the clinical microbiology laboratory for *C. difficile* testing. Specimens were tested for the presence of TcdA by immunoassay (Clearview *C. difficile* Toxin A EIA, Wampole Laboratories, Cranbury, NJ, USA) and for the presence of *C. difficile* by culture.

#### 2.1.1. Culture

All stool samples were cultured anaerobically for *C. difficile* on selective cycloserine cefoxitin fructose agar (CCFA) media as previously described [25]. Colonies of *C. difficile* identified on culture were inoculated into Chopped Meat Carbohydrate Medium (CMCM) and incubated at 35 degrees C for 48 h. 

#### 2.1.2. In Vitro Testing of Recovered *C. difficile* Isolates for TcdA and Cytotoxicity

**Figure 1:**
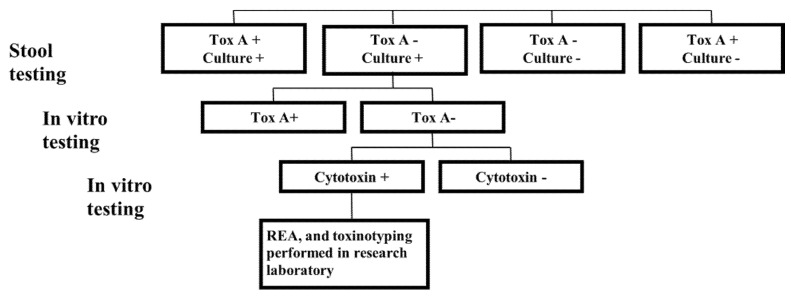
Stool *C. difficile* testing protocol using TcdA (Tox A) immunoassay and culture from 1 September 1999 to 1 February 2002.

*C. difficile* isolates that were recovered from stool samples but were negative for TcdA by EIA in the clinical laboratory were subjected to in vitro testing for TcdA. Supernatant of the CMCM was diluted 1:10 and tested by TcdA EIA as per the manufacturer’s directions (*C. difficile* TOX-A, TechLab, Blacksburg, VA, USA). Isolates that were negative for TcdA underwent a qualitative cytotoxin analysis. *C. difficile*-inoculated CMCM was diluted 1:10 and aliquots were pre-incubated with either diluent or *C. difficile* antitoxin. Each separate aliquot was inoculated onto cell cultures of human foreskin fibroblasts (final dilution 1:200) (Toxi-Titer cytotoxicity assay, Trinity Biotech, Carlsbad, CA, USA). Cells were screened for cytopathic changes at 24 and 48 h. Supernatant aliquots that demonstrated cytotoxicity at the final titer (1:200) and which were neutralized by the antitoxin were considered positive. Supernatants from known toxigenic and non-toxigenic strains were included as the positive and negative controls, respectively. *C. difficile* isolates that were negative by TcdA EIA but had a positive cytotoxicity assay underwent REA typing and toxinotyping in our research laboratory (Figure 1). 

### 2.2. Research Laboratory Testing

#### 2.2.1. Restriction Endonuclease Analysis (REA) Typing

*C. difficile* isolates were cultured in brain heart infusion or tryptic soy broth (TSB). Using methods previously described by Clabots et al., DNA was isolated, purified, and subjected to *Hin*dIII restriction enzyme digestion and electrophoresis on 0.7% agarose gel [25]. The pattern of each isolate was visually compared with a known database [26,27]. Patterns that had a 90% similarity index were placed within the same REA group (letter designations) and identical patterns were classified by a subgroup (number designation).

#### 2.2.2. Toxinotyping

*C. difficile* strains were inoculated into TSB broth from a blood agar plate 48-h culture and incubated anaerobically overnight. Total cellular DNA was isolated as previously described [25]. Polymerase chain reaction (PCR) was performed to create fragments A3 and B1, which were subsequently restricted by *Hin*cII or *Acc*I (B1) and *Eco*RI (A3) and compared to reference strains as described by Rupnik et al. [7].

### 2.3. Clinical Data

A chart review of patients with isolates determined to be TcdA−/B+ was conducted to obtain demographics, medications, CDI treatment, and outcomes. The determination of initial versus recurrent CDI, healthcare associated CDI (HA-CDI), and community associated CDI (CA-CDI) were classified per the Infectious Diseases Society of America and Society for Healthcare Epidemiology of America (IDSA/SHEA) guidelines [5]. The institutional review board of the hospital approved the study protocol. 

## 3. Results

### 3.1. Surveillance by TcdA Immunoassay

From 1 September, 1999 to 1 January, 2002, 10,668 stools were submitted for *C. difficile* testing. In total, 1661 of the stool specimens were positive by Toxin A EIA or culture (15.6%, 95% Confidence Interval (CI): 14.9–16.3%). Of these 1661 C. difficile-positive stool specimens, *C. difficile* culture and TcdA EIA were positive in 1436 (86.5%, 95% CI: 84.8–88.1%) and 586 (35.3%, 95% CI: 32.9–37.6%), respectively (Table 1). Among the *C. difficile* 1436 stools that were culture positive, 1075 were TcdA EIA negative in the clinical laboratory (74.9%, 95% CI: 72.6–77.1%). 

Our reference laboratory documented in vitro TcdA production in 786 of the 1075 isolates (73.1%, 95% CI: 70.4–75.8%). Of the remaining isolates, 289 (26.9%, CI:24.3–29.6%) were negative for TcdA by in vitro analysis. Further analysis of the 289 in vitro TcdA−negative isolates revealed that 23 isolates were positive by in vitro cytotoxicity assay indicating the presence of TcdB. Therefore, 1.6% (23/1436) of the isolates that were recovered during this 2.5-year surveillance period were TcdA−/B+ variants (Table 1).

### 3.2. Molecular Analysis of TcdA−Negative, B-Positive Variant Isolates

**Figure 2:**
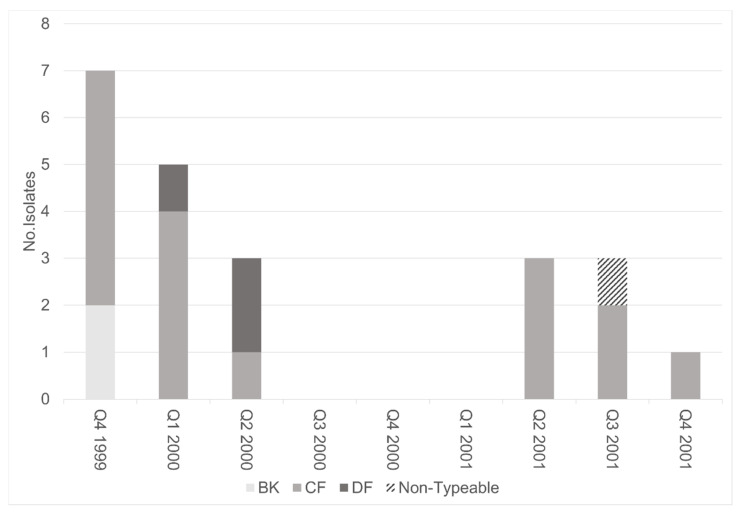
Epidemiologic curve of all TcdA−/B+ variant isolates and (B) restriction endonuclease analysis (REA) group CF isolates per yearly quarter (3 month time interval: Q1, Q2, Q3, Q4). Among REA group CF, 50% (8/16) were the specific REA type CF4. REA CF4 was found throughout the study period. Other CF types included: CF2, CF6, CF7, CF8, CF9, CF10, CF18, CF19 (1 isolate each).

**Figure 3:**
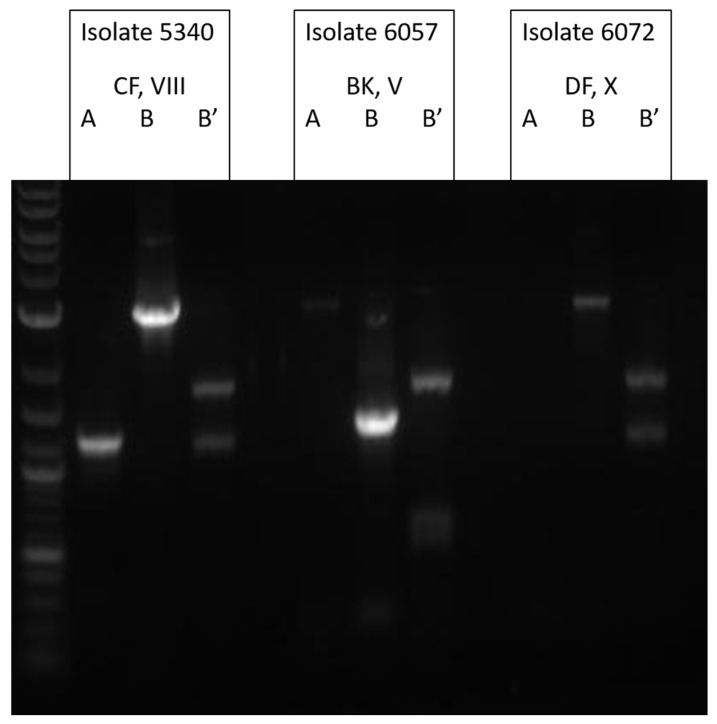
Toxinotyping of toxin variant strains of *C. difficile*. Listed by isolate number, REA type and toxinotype. “A” represents the A3-PCR fragment digested with EcoRI, “B” is the B1-PCR fragment digested with HincII and “B’” is the B1-PCR fragment digested with AccI. Shown here is the previously unreported REA type DF strain, identified as possibly similar to toxinotype X, but we were unable to amplify the A3 fragment.

Twenty-two TcdA−/B+ variant isolates were characterized by REA and representative isolates were analyzed by toxinotyping (one isolate was unrecoverable after initial testing). These variants were found throughout the study time period with no obvious clustering (Figure 2). The majority of toxin variants examined belonged to REA group CF (73%, 16/22). Eight of these group CF isolates were the specific REA type, CF4. The other 8 CF isolates were identified as eight different REA subgroups (Figure 2). This strain was previously identified as the cause of a fatal case of CDI in our institution [24]. Toxinotyping of a representative CF isolate confirmed this strain to be toxinotype VIII (Figure 3), consistent with our previous analyses of REA group CF strains [28].

In addition to the REA group CF strains, we identified two new TcdA-/B+ variants. The restriction pattern of three isolates had never been seen before at the time of diagnosis and was designated as a new REA group (DF) and REA type, DF 1. The toxinotype pattern of this isolate was consistent with toxinotype X (Figure 3). Two isolates were identified as REA group BK and toxinotype V (Figure 3).

### 3.3. Clinical Outcomes

The 23 TcdA−/B+ variant isolates were recovered from 21 patients whose clinical charts were reviewed for clinical management and outcome (Table 2). Among these patients, 48% (10/21) were female and the median age was 60 years (range: 37–86). The initial CDI episode was healthcare facility-associated in 71% (15/21) of the cases. Recent antibiotic exposure was documented in 84% (16/19) and diarrhea was noted in 95% (19/20). Less than half of the patients (37%, 7/19) received specific therapy for CDI and the symptoms in patients who did not receive specific therapy resolved spontaneously. There were no deaths and no other identified complications.

## 4. Discussion

From September 1999 to January 2002, TcdA−/B+ variants accounted for 1.6% of all the recovered *C. difficile* isolates within a single healthcare system in United States. The majority of these isolates were identified as REA group CF, a well-characterized TcdA−/B+ *C. difficile* variant [13,14]. We also found two additional REA groups, BK and DF, which were also identified as TcdA−/B+ isolates. 

REA group CF commonly correlates with PCR-Ribotype 017 (RT017), toxinotype VIII, NAP9, and ST37 [28,29,30,31] and has been well established as a TcdA−/B+ variant that results in a symptomatic CDI through the actions of toxin B [32]. CF/RT017 has been found worldwide but is endemic to East Asia and historically accounted for 15–40% of CDI in South Korea, Taiwan, and China [33,34,35]. Initial studies indicated that CF/RT017 was capable of causing severe disease and even mortality [21,22,23,24,28,32,33,34,35,36]. Within our own healthcare system, we reported a death due to REA group CF related CDI [24]. The outcome of this case was likely attributed to a lack of specific antibiotic treatment due to an overreliance on clinical laboratory testing which at the time only included toxin identification by TcdA EIA, despite overwhelming clinical evidence of CDI [24]. Severe outcomes due to TcdA−/B+ CDI are well documented, particularly in outbreak settings, however, the full spectrum of infection with these variants is not clear, particularly in certain regions of Asia where RT 017 is common and where severe disease and mortality are uncommon [32]. Data provided by our study would further support that CDI caused by TcdA−/B+ variants more commonly results in less severe CDI. There were no reported fatal events within our study and the diagnoses and treatments were often delayed in subjects with TcdA−/B+ related CDI due to the delay in final test results which involved culture and in vitro toxin testing of the recovered isolate. In fact, the final test results were not reported in some cases until after the patient had improved and was discharged from the hospital.

Over the past three decades, the understanding of TcdA and TcdB has expanded greatly. Initial animal models using purified TcdA and TcdB indicated that TcdA was the primary virulence determinant [10,37]. As such, emphasis was placed on TcdA for the detection of *C. difficile* within clinical laboratories [8]. However, subsequent animal experiments using isogenic TcdA and TcdB *C. difficile* mutants demonstrated that TcdB is not only sufficient in causing disease, but also causes CDI through multiple mechanisms [11,12,38]. Moreover, Carter, et al. used isogenic *C. difficile* mutants in three different animal models, showing that the TcdA−/B+ mutants were attenuated in virulence [39]. Furthermore, TcdB alone caused severe localized intestinal damage as well as systemic organ damage and TcdB was primarily responsible for inducing host innate and inflammatory immune responses [39]. 

These data supporting the importance of TcdB are further bolstered by the clinical observations of TcdA−/B+ isolates resulting in CDI. Additionally, the importance of TcdB is also supported by a randomized clinical trial using the monoclonal bezlotoxumab [40,41]. Bezlotoxumab, a monoclonal antibody directed towards TcdB, has demonstrated the capacity to decrease recurrent CDI through binding TcdB [40,41]. However, no such effect was noted with the monoclonal antibody with activity towards TcdA [40]. As such, the primary focus in both clinical and molecular epidemiology has focused more so on TcdB.

Recent data have demonstrated that TcdA causes cellular apoptosis in a manner distinctly different than TcdB [38]. TcdA, a potent enterotoxin, has been shown to induce epithelial apoptosis through glucosyltransferase-dependent caspase-3 activation [38]. This action by TcdA in turn results in epithelial tight junction disruption and likely results in mucosal damage and fluid loss [42]. TcdB, a cytotoxin, causes cellular necrosis and apoptosis via a glucosyltransferase-independent reactive oxygen species production [38,43]. Despite these differences, toxin A+/B- mutants have demonstrated virulence in vitro, albeit with somewhat attenuated virulence [12,39]. Further data supporting the importance of TcdA can be seen in animal models. Hamster models have demonstrated that while TcdA−/B+ REA CF strains are virulent, they also demonstrate delayed colonization and incomplete mortality when compared with fully toxigenic (A+/B+) strains [44]. These findings support roles for both toxin A and B in inducing a CDI as well as colonization.

The studies surrounding REA group CF (RT017) reveal that TcdA−/B+ variants are clearly pathogenic [21,22,23,24,28,32,33,34,35,36]. However, a detailed clinical study to determine the clinical outcomes and the virulence of this group strain is lacking. Our data, while limited to one institution, indicate that these variant isolates may not carry the same risk of severe CDI as fully toxigenic A+/B+ isolates. Our study was limited by the lack of a contemporary control group comparing clinical outcomes in patients infected with toxin A+/B+ strains. However, we included all patients identified with TcdA−/TcdB+ over a 2.5-year period in our institution and did not identify any severe or complicated infections. 

Moreover, while CF/RT017 is more common in Asia, our study reveals that REA group BK (RT078/128), a strain more common in Europe, can lack TcdA [45]. Current data surrounding REA group BK (RT078/128) support the hypothesis that the group is a fully toxigenic and harbors both TcdA and TcdB [46]. Our findings indicate that group strains which we presume to be fully toxigenic may harbor mutations which render them TcdA−/B+ variants, yet remain virulent [30]. To fully understand the implications of *C. difficile* TcdA−/B+ variant isolates, robust genomic and molecular epidemiologic studies will be required to answer these still yet unanswered questions regarding the importance of *C. difficile* toxins A and B.

## Figures and Tables

**Table 1:** C. difficile testing results on submitted stool specimens and recovered *C. difficile* isolates.

Testing Modality	All Stools (*n* = 10,668)	95% Confidence Interval
TcdA EIA positive OR Culture positive	1661 (15.6%)	14.9–16.3%
	Culture- or toxin A EIA-positive stools (*n* = 1661)	
Culture positive	1436 (86.5%)	84.8–88.1%
TcdA EIA positive	361 (25.1%)	22.9–27.4%
TcdA EIA negative	1075 (74.9%)	72.6–77.1%
TcdA EIA positive	586 (35.3%)	32.9–37.6%
Culture negative	225 (38.4%)	34.5–42.3%
	Recovered *C. difficile* isolates from culture-positive/EIA-negative stools (*n* = 1075)	
In vitro TcdA positive	786 (73.1%)	70.4–75.8%
In vitro TcdA negative	289 (26.9%)	24.3–29.6%
In vitro cytotoxicity assay positive	23 (8.0%) *	5.1–11.7%

EIA: Enzyme immunoassay.* 8.0% of isolates that were negative for TcdA in vitro (23/289) or 1.6% of all culture-positive stools were TcdA−/B+ (23/1436).

**Table 2:** Clinical characteristics * of patients with TcdA−/B+ variant *C. difficile* infection.

Median Age (Range)	60 (37–86)
Female (*n*)	47.6% (10/21)
HA-CDI (*n*)	71.4% (15/21)
Prior Antibiotics (*n*)	84.2% (16/19)
Diarrhea (*n*)	95% (19/20)
Treated for CDI (*n*)	36.8% (7/19)

* Clinical data were not available for all 21 patients.

## Data Availability

The data presented in this study are available on request from the corresponding author. The data are not publicly available due to privacy and ethical concerns.
